# Plasma Proteomic Profiling of Young and Older Adults Identifies Candidate Biomarkers of Biological Aging at the Intersection of Age and Disease

**DOI:** 10.1111/acel.70469

**Published:** 2026-04-03

**Authors:** Juliette Tavenier, Nikolaj Normann Holm, Thomas Kallemose, Morten Baltzer Houlind, Aino Leegaard Andersen, Line Fleischer Hach, Magnus Berglind, Ove Andersen, Jan O. Nehlin, Line Jee Hartmann Rasmussen

**Affiliations:** ^1^ Department of Clinical Research Copenhagen University Hospital Amager and Hvidovre Hvidovre Denmark; ^2^ Department of Applied Mathematics and Computer Science Technical University of Denmark Kongens Lyngby Denmark; ^3^ The Capital Region Pharmacy Herlev Denmark; ^4^ Department of Drug Design and Pharmacology University of Copenhagen Copenhagen Denmark; ^5^ Department of Clinical Medicine, Faculty of Health and Medical Sciences University of Copenhagen Copenhagen Denmark; ^6^ The Emergency Department Copenhagen University Hospital Amager and Hvidovre Hvidovre Denmark; ^7^ Department of Psychology & Neuroscience Duke University Durham North Carolina USA

**Keywords:** aging, biological aging, biomarkers, chronic disease, plasma proteome, senescence

## Abstract

Aging and chronic diseases intersect at the level of biological aging mechanisms, where age‐related molecular and cellular changes contribute to the development of diverse pathologies. Biomarkers of biological aging could help predict and track the progression of chronic diseases and evaluate the effectiveness of interventions aimed at promoting healthy aging. Here, we aimed to identify biomarkers reflecting biological aging by analyzing protein signatures shared between older age and elevated disease burden. Using the Olink Explore HT platform, we measured 5416 plasma proteins in 52 recently hospitalized Older Patients (≥ 65 years), 52 age‐ and sex‐matched Older Controls, and 20 healthy Young Controls (20–25 years). We identified 797 proteins that differed with chronological age group by comparing Older and Young Controls, and 761 proteins that differed with disease burden by comparing Older Patients and Older Controls. Of these, 311 proteins were differentially expressed across both chronological age and disease burden comparisons and were defined as biological Aging Proteins (APs). We compared the identified APs with findings from prior proteomic studies of aging and disease to uncover previously unreported proteins associated with biological aging. Unsupervised hierarchical clustering analysis of the 5416 proteins revealed eight clusters based on expression patterns, one significantly enriched for APs, suggesting shared regulatory pathways. Our findings highlight known and novel plasma biomarkers associated with biological aging, with potential utility for risk stratification and the development of interventions targeting the aging process.

## Graphical abstract

By comparing plasma proteomes of young and older adults, as well as older adults with varying levels of disease burden, this study aims to identify plasma proteins at the intersection of age and disease. The identified signature points to potential candidate biomarkers of biological aging.

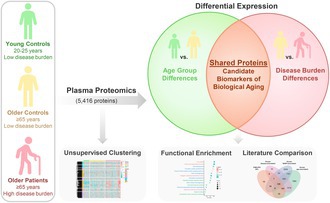

## 1 Introduction

The global increase in life expectancy is paralleled with the increasing prevalence of chronic diseases and multimorbidity, placing growing demands on healthcare systems worldwide for long‐term care and disease management (Barnett et al. 2012; GBD 2019 Ageing Collaborators 2022). The geroscience hypothesis proposes that aging biology is a fundamental driver of most chronic diseases, including cardiovascular diseases, type 2 diabetes, cancer, and neurodegenerative disorders (Sierra 2016). This framework also suggests that targeting aging mechanisms could simultaneously delay or prevent multiple conditions (Kennedy et al. 2014). Hallmarks of aging, such as cellular senescence and chronic inflammation, are increasingly recognized as contributors to disease and multimorbidity, and represent promising targets for intervention (López‐Otín et al. 2023; Morsli and Bellantuono 2021; Tartiere et al. 2024). For example, senescent cells and their senescence‐associated secretory phenotype (SASP) are being explored as clinical biomarkers for assessing medical risk and mortality, and as targets for intervention with senotherapeutics (Schafer et al. 2020; St Sauver et al. 2023). Identifying and validating reliable biomarkers of biological aging could enable early identification of individuals at elevated risk of chronic diseases, facilitating personalized medicine approaches based on biological age stratification. This could support timely interventions to address health disparities and improve overall population health (Elliott et al. 2021; Garmany et al. 2021).

Circulating plasma proteins are promising biomarkers due to their accessibility and consistent correlations with chronological age (Coenen et al. 2023; Lehallier et al. 2019; Sayed et al. 2021; Tanaka et al. 2018). However, distinguishing biomarkers of biological aging reflecting the multisystem decline in physiological integrity that occurs with advancing age from those that simply correlate with chronological age remains a major challenge. Most proteomic studies to date have used generally healthy cohorts to identify proteins that change with age, often overlooking individuals with multiple chronic age‐related diseases likely to exhibit accelerated biological aging. These studies do not allow one to distinguish proteins that simply mark age‐related physiological changes without contributing to disease development from those playing causal roles in pathology. Conversely, disease‐focused studies often exclude individuals with comorbidities (multimorbidity), despite such individuals representing the majority of older adults (Barnett et al. 2012; Schiøtz et al. 2017), and typically lack healthy young controls. As a result, these studies fail to account for age as a modulating factor, making it difficult to determine whether observed protein changes reflect disease‐specific pathology, aging‐related processes, or both. By focusing on individual diseases, these also overlook the broader role of aging as a fundamental driver of pathological processes across multiple organ systems. This limits our understanding of how aging and disease biology intersect, as not all proteins that change with age contribute to disease, and vice versa. Instead, proteins whose expression and function change with both age and disease status may better capture the shared biological mechanisms underlying aging and chronic diseases, serving as biomarkers of biological aging that may not only track, but also contribute to, age‐related physiological decline, offering potential not only as biomarkers, but also as targets for intervention.

**FIGURE 1:**
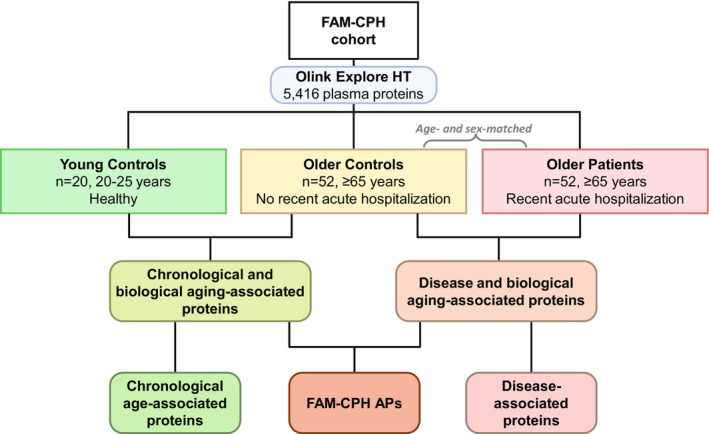
Study design for the FAM‐CPH cohort. Expression levels of 5416 plasma proteins were assessed using high‐throughput Olink proximity extension assay (PEA)‐based proteomics. Proteins associated with chronological age groups are identified by comparing Older Controls to Young Controls. Proteins associated with disease burden are identified by comparing Older Patients to age‐ and sex‐matched Older Controls. Proteins associated with both chronological age groups and disease burden are considered potential biomarkers of biological aging: FAM‐CPH Aging Proteins (FAM‐CPH APs).

To identify proteins at the intersection of age and disease, we conducted exploratory proteomics within a cohort designed to investigate biological aging, assessing age‐related differences in protein expression by comparing healthy Young Controls to Older Controls with low disease burden, and disease‐related differences by comparing these Older Controls to age‐ and sex‐matched Older Patients with a recent acute hospitalization, indicative of higher disease burden and physiological dysregulation (Figure 1). In this cohort, we previously showed that Older Patients follow a different health trajectory than their age‐matched peers, exhibiting signs of advanced biological aging, including elevated systemic chronic inflammation, SASP proteins, immune dysfunction, and physical and cognitive decline (Tavenier, Rasmussen, et al. 2021; Tavenier et al. 2020). Thus, this design enables us to identify proteins that differ by age group, by disease burden, and those shared between these comparisons, which may provide insight into biological aging. In addition, the use of an unselected patient population with diverse chronic diseases, including cardiovascular, renal, cerebrovascular diseases, and diabetes, further enhances our ability to identify proteins reflecting shared aging pathways rather than disease‐specific pathways.

This study aims to (1) identify plasma protein signatures showing overlap between older age and elevated disease burden as candidate markers of biological aging; (2) compare these with previously reported age, disease, or SASP‐associated proteins to identify previously unrecognized biomarkers of biological aging; and (3) identify a set of proteins associated with mortality risk (see Figure S1 for an overview of the analyses). We differentiate between markers of chronological age: correlated with age but without disease relevance; markers of disease processes: reflecting pathological processes, which may become more prevalent or severe with age, but are not necessarily markers of aging itself; and markers of biological aging: correlated with both chronological age and increased disease burden, and which may actively contribute to age‐related decline and disease. Our focus is on this latter group, which may hold the greatest promise for clinical application, both to monitor biological aging and as therapeutic targets.

## 2 Methods

### 2.1 Study Design and Participants

This study is a secondary analysis using data from a subpopulation within the FAM‐CPH cohort (“Fælles Akutmodtagelse” [in English: the Joint Emergency Department] Copenhagen) established in the years preceding the Danish national reform that introduced joint Emergency Departments (ED) staffed with physicians from multiple specialties. FAM‐CPH is a prospective longitudinal observational study aiming to provide insight into patient characteristics and investigate mechanisms of biological aging, chronic inflammation, malnutrition, and medication use in acutely ill older adults, older adults without recent hospitalization, and healthy young adults prior to the reform (clinicaltrials.gov: NCT03052192). The study design and results have previously been described (Andersen et al. 2021; Bornæs et al. 2022; Hansen et al. 2021; Houlind et al. 2020; Tavenier, Rasmussen, et al. 2021; Tavenier et al. 2020). The study protocol was approved by the Health Research Ethics Committee for the Capital Region of Denmark (ref.# H‐16038786) and the Danish Data Protection Agency (ref.# AHH‐2016‐067). The study was conducted in accordance with the Declaration of Helsinki. All participants gave written informed consent.

The FAM‐CPH cohort consists of 128 Older Patients, 52 Older Controls, and 59 Young Controls. This study uses data from a subset of participants: (1) Older Patients (*n* = 52): adults aged ≥ 65 years, admitted to the ED at Copenhagen University Hospital Hvidovre for acute medical illness between November 15th, 2016, and July 26th, 2017. The primary reason for hospital admission was categorized as: cardiovascular symptoms, infections, respiratory symptoms, falls, or other, based on the patient's self‐reported symptoms at the time of ED admission. Data and samples used in this study were collected during a follow‐up visit 30 days after discharge from the acute admission to allow for recovery from acute illness. (2) Older Controls (*n* = 52): adults aged ≥ 65 years without acute hospital admission within 2 years prior to inclusion, matched 1:1 based on age and sex with Older Patients at the 30‐day follow‐up, and were included between February 6th, 2018, and November 5th, 2018. (3) Young Controls (*n* = 20): healthy individuals aged 20–25 years, included between September 28th, 2019, and October 14th, 2019. The 10 youngest men and 10 youngest women were selected for this study from a larger group of 59 Young Controls. All participants were followed until October 2nd, 2024, when information on survival status and date of death was extracted from the participants' electronic health records.

### 2.2 Plasma Proteomics

Blood samples were collected by venipuncture in EDTA tubes and centrifuged for plasma isolation. The plasma was aliquoted and stored at −80°C until analysis. The relative expression of 5416 proteins was measured using the Olink Explore HT platform (Olink, Uppsala, Sweden) by BioXpedia A/S (Aarhus, Denmark), an Olink‐certified contract research laboratory, according to the manufacturer's instructions. Samples from the three different groups were randomized and evenly distributed on the two plates, and samples from matched case–control pairs were analyzed on the same plate. Olink Explore HT uses Proximity Extension Assay (PEA) technology, where multiplexed oligonucleotide‐labeled antibody probe pairs bind to their respective target protein in the sample (Wik et al. 2021). When the antibody pairs bind in proximity, the matched oligo sequences hybridize, forming a PCR reporter sequence through a DNA polymerization event. The DNA barcode is then amplified using PCR, which is subsequently detected and quantified using Next Generation Sequencing (NGS) readout. Internal and external controls are used for data normalization and quality control. Finally, the protein expression level is calculated using dedicated processing software, and results are reported in the relative expression unit NPX (Normalized Protein Expression) on a log2 scale. No samples or proteins were removed due to a major QC flag.

### 2.3 Blood Biomarkers

Plasma levels of inflammatory markers and standard blood biochemistry markers were quantified as previously described (Tavenier, Rasmussen, et al. 2021; Tavenier et al. 2020). Briefly, inflammatory biomarker levels were measured in EDTA plasma: IL‐6 and TNF‐α levels using a magnetic Human High Sensitivity Luminex Performance assay (R&D Systems, Minneapolis, MN, USA), and IL‐18 using a magnetic Human Luminex Discovery assay (R&D Systems), on the Luminex 200 System (Luminex Corporation, Austin, TX, USA); GDF15 using a Human Quantikine ELISA assay (R&D Systems); and suPAR using a suPARnostic ELISA assay (ViroGates A/S, Birkerød, Denmark). Routine biochemistry included: alanine aminotransferase, albumin, alkaline phosphatase, bilirubin, blood urea nitrogen, coagulation factors II, VII and X, C‐reactive protein [CRP], creatinine, hemoglobin, lactate dehydrogenase, mean corpuscular hemoglobin concentration, mean corpuscular volume, neutrophils, potassium, sodium, thrombocytes, white blood cell count, cystatin C, total cholesterol, high‐density lipoprotein (HDL), low density lipoprotein (LDL), triglycerides, and glycated hemoglobin (HbA1c), and were measured using established clinical methods at the Department of Clinical Biochemistry, Copenhagen University Hospital Hvidovre.

### 2.4 Physiological Measures

Anthropometric data included body mass index (BMI; kg/m^2^) derived from height and weight measurements, and waist circumference measured in the standing position at the level of the navel. Blood pressure was measured in a seated position at rest using a standard automated sphygmomanometer.

Frailty was assessed using the frailty index (FI)‐OutRef, a routine biochemistry biomarker‐based index developed in an acute care setting (Klausen et al. 2017). The index is derived from 17 routine blood biochemistry markers, with one point assigned for each marker measurement falling outside its reference range. The total score reflects the cumulative burden of physiological dysregulation, with higher values indicating greater frailty.

### 2.5 Functional Assessments

Physical and cognitive function were assessed as previously described (Tavenier, Rasmussen, et al. 2021; Tavenier et al. 2020). Physical function measures included hand grip strength, the 30‐s chair stand test, and 4‐m gait speed. Cognitive function was evaluated using the Symbol Digit Modalities Test (SDMT) (Smith 1982), Trail Making Test‐A (TMT‐A) and ‐B (TMT‐B) (Bowie and Harvey 2006), and the total recall score from the Hopkins Verbal Learning Test—Revised (HVLT‐R) (Benedict et al. 1998).

### 2.6 Statistics

For descriptive statistics, continuous data are presented as median and interquartile range (IQR) or mean and standard deviation (SD) when normally distributed. Categorical data are presented as *n* (%). All *p* values < 0.05, either raw or corrected for multiple comparisons, are considered statistically significant. All analyses were performed using R version 4.1 (R Core Team 2021) using packages *cluster* (Maechler et al. 2025), *ClusterProfiler* (Wu et al. 2021), *glmnet* (Friedman et al. 2010; Simon et al. 2011), and *survival* (Therneau 2024). Figures were created with R using the *ggplot2* (Wickham 2016) and *pheatmap* (Kolde 2025) packages and Microsoft PowerPoint.

Differential expression analyses were conducted using two pairwise comparisons: between Older Controls and Young Controls, and between Older Patients and Older Controls, using either *t*‐tests for independent samples or paired samples, as required by the study design. Before conducting the tests, it was first checked if distributions could be assumed to be normally distributed using the Shapiro–Wilk test. If this was not the case, a Wilcoxon rank sum test (non‐paired data) or signed rank test (paired data) (Whitley and Ball 2002) was conducted instead according to the study design. Furthermore, the fold change was calculated on a linear scale as the geometric mean of the first group divided by the geometric mean of the second group. The *p* values were corrected for multiple testing using the Benjamini‐Hochberg method (Benjamini and Hochberg 1995). Differential protein expression for each comparison was visualized using volcano plots displaying the relationship between fold changes and *p* values for all 5416 proteins for each comparison. Proteins associated with biological aging (FAM‐CPH APs) were identified based on our methodological rationale as proteins that were differentially expressed in both comparisons. This approach aimed to capture proteins that differed by both chronological age group and disease burden, potentially reflecting early or subclinical processes of age‐related disease (biological Aging‐Associated FAM‐CPH APs), while distinguishing them from those solely differing between chronological age groups (Chronological Age‐Associated) and from those related to the presence of established disease differing only between disease burden groups (Disease‐Associated). The number of overlapping proteins differentially expressed between the two comparisons was visualized using a Venn diagram. Pearson's correlation coefficients with 95% confidence intervals (CIs) were calculated to test associations between biomarker levels measured with the PEA technology (Olink Explore HT) and immunoassays (ELISA and Luminex).

To evaluate the novelty and reproducibility of our findings, we compared the proteins identified in our analyses with those reported in previously published human plasma or serum proteomic studies. Specifically, we cross‐referenced the proteins from our dataset: Chronological Age‐Associated, Disease‐Associated, and biological Aging‐Associated (FAM‐CPH APs) proteins against previous proteomic studies reporting protein associations with (a) age, (b) disease, or (c) the SASP. We identified relevant studies through a targeted literature review in PubMed, focusing on studies that utilized targeted large‐scale proteomic platforms (SomaScan or Olink), prioritizing those with broad protein coverage, and reporting associations with chronological age, diseases and geriatric syndromes (frailty or mobility disability). In total, we included 19 studies reporting on age‐associated proteins (Argentieri et al. 2024; Coenen et al. 2023; Evans et al. 2024; Goeminne et al. 2025; Lehallier et al. 2019; Lind et al. 2019; Menni et al. 2015; Moaddel et al. 2025; Mörseburg et al. 2025; Oh et al. 2025, 2023; Sathyan, Ayers, Gao, Weiss, et al. 2020; Sun et al. 2023; Tanaka et al. 2020, 2018; Tin et al. 2019; Wang, Huang, et al. 2025; Wang et al. 2024; Wang, Xiao, et al. 2025) and 16 studies reporting on disease‐, physical decline‐, or frailty‐associated proteins (Carrasco‐Zanini et al. 2024; Gadd et al. 2024; Kuo et al. 2024, 2025; Landino et al. 2020; Liu et al. 2023, 2024; Ma et al. 2025; Mitchell et al. 2023; Osawa et al. 2020; Sathyan, Ayers, Gao, Milman, et al. 2020; Sathyan et al. 2025; Ubaida‐Mohien et al. 2025; Verghese et al. 2021; Xu et al. 2025). In addition, we identified 12 studies identifying factors secreted as part of the SASP (Acosta et al. 2013; Alessio et al. 2023; Basisty et al. 2020; Boroumand et al. 2025; Coppé et al. 2010, 2008; Griukova et al. 2019; Lasry and Ben‐Neriah 2015; Olinger et al. 2025; Özcan et al. 2016; Schafer et al. 2020; Wiley et al. 2019). For each publication, we extracted the relevant protein lists, including full sets of proteins reported to be significantly associated with, or predicting, chronological age or disease, as well as any reduced panels, predictive protein signatures, or “core” proteins highlighted by the authors. We quantified the overlap between each literature‐derived list and the full Olink HT panel, as well as each of the three categories identified in our study. Overlaps were verified using UniProt identifiers and reported as the number and percentage of shared proteins between each literature list and each of our categories. In addition, literature‐derived lists of known age‐, disease‐, or SASP‐associated proteins were aggregated and the overlap between each aggregated list, as well as with the FAM‐CPH APs, was evaluated to identify proteins consistently reported across studies and to highlight potentially novel biomarkers uniquely associated with biological aging in our dataset.

We performed unsupervised cluster analysis using all 5416 proteins to identify groups of proteins based on similar expression across the three participant groups. To ensure equal contribution from each protein in the clustering, we computed *z*‐scores for each protein individually based on NPX values. We performed agglomerative hierarchical clustering analysis using the *hclust* function from the *cluster* package in R with complete linkage and Euclidean distance as dissimilarity measure. The cutoff was determined based on the scree plot of the dendrogram agglomerations (Figure S4). Results are visualized as dendrograms and associated heatmaps. To ascertain the stability of the clustering, we randomly sampled a proportion (0.85, 0.90, and 0.95) of the population 100 times, clustering each sample and comparing the protein partitioning in the subsample with the full sample clustering by the Rand index (Rand 1971). To adjust for the trivial scaling of the Rand Index with increasing cluster sizes, a normalized stability score was estimated using normalization by random labels (Figure S5) (von Luxburg 2010). As an additional sensitivity analysis, we conducted similar analyses using the Manhattan distance (see Figure S6). The mean relative expression of the proteins within each cluster, stratified by group, was visualized using boxplots displaying median and interquartile range, and whiskers extending to 1.5 times the IQR. Additionally, dot plots and violin plots were overlaid onto the boxplots to display the individual data points and provide an approximate distribution of the data. To test for enrichment of Chronological Age‐, Disease‐, or biological Aging‐Associated Proteins among the clusters, hypergeometric enrichment tests were performed based on the total number of identified proteins in each category, out of all measured proteins (5416 proteins). Obtained *p* values were corrected using the Benjamini‐Hochberg method.

To evaluate the biological relevance of the identified groups of proteins, we performed over‐representation analysis (ORA) using hyper geometric tests. The gene sets for the following databases were downloaded from https://www.gsea‐msigdb.org/gsea/msigdb/collections.jsp and stacked as one dataset: Gene Ontology (GO; biological process, molecular function, and cellular component), Reactome, KEGG (Kyoto Encyclopedia of Genes and Genomes), and Hallmark. The full list of 5416 proteins in the Olink Explore HT panel was used as the background protein set, and all databases were stacked into one dataset for the analysis. Analyses were performed separately for Chronological aging‐associated proteins, Disease‐associated proteins, FAM‐CPH APs, and cluster 8 proteins. *p* values were corrected using the Benjamini‐Hochberg method. The top 15 pathways for each protein group are reported.

Regularized Cox regression with a LASSO penalty was used to analyze associations between FAM‐CPH APs and time‐to‐death. Data from Young Controls were not included in these analyses as none had died during follow‐up. The model included unpenalized adjustment for sex, and the baseline hazard was stratified by group. The optimal value of the penalization parameter was determined using 10‐fold cross‐ validation by minimizing the partial likelihood deviance, and nonzero aging protein coefficients were selected. As a robustness criterion, we repeated the selection procedure across 1000 stratified bootstrap samples, each consisting of 52 Older Patients and 52 Older Controls drawn with replacement. Proteins selected in at least 50% of the bootstrap samples were deemed “robustly selected”. Pearson correlation analyses were performed to assess the relationship between the NPX values of the identified proteins and measures of inflammation, biochemistry, and metabolism, as well as frailty, and physical and cognitive function.

## 3 Results

### 3.1 The FAM‐CPH Cohort

We conducted a plasma proteomic analysis on a sub‐sample of the FAM‐CPH cohort, including 20 Young Controls (median age 23 years, 50% female), 52 Older Controls, and 52 age‐ and sex‐matched Older Patients (median age 75 years, 48% female), Figure 1 and Table 1. For Older Patients, blood samples were collected 30 days after hospital discharge, to allow recovery from the acute illness and better capture their habitual health state. The Older Patients had been admitted for a wide range of clinical indications, including cardiovascular symptoms (30.8%), infection (21.2%), respiratory symptoms (19.2%), falls (9.6%), or other reasons (19.2%), and 65.5% had several pre‐existing chronic conditions prior to admission. Older Controls exhibited higher levels of systemic inflammation and physical and cognitive decline compared to Young Controls. These impairments, along with increased frailty, were further exacerbated in Older Patients compared to their age‐matched Older Controls. The study participants were followed for a median of 6.54 years (median [range]: Older Patients 7.00 years [0.16–7.79]; Older Controls 6.58 years [0.53–6.66]). Older Patients exhibited higher mortality (55.8%) than their age‐matched Controls (7.7%) during the follow‐up period.

**TABLE 1:** Baseline characteristics of the study participants.

	*n*	Young Controls (*n* = 20)	*n*	Older Controls (*n* = 52)	*n*	Older Patients (*n* = 52)
Demographics and lifestyle						
Age (years)	20	23.2 (21.9–24.2)	52	74.8 (70.7–81.9)	52	74.9 (70.7–82.0)
Sex (female)		10 (50.0%)	52	25 (48.1%)	52	25 (48.1%)
Smoking	20		52		52	
Daily smoker		0 (0.0%)		4 (7.7%)		6 (11.5%)
Occasional smoker		2 (10.0%)		0 (0.0%)		0 (0.0%)
Former smoker		6 (30.0%)		29 (55.8%)		34 (65.4%)
Never smoker		12 (60.0%)		19 (36.5%)		12 (23.1%)
Clinical measures						
Waist circumference (cm)	20	73.5 (70.0–78.5)	52	96.5 (88.0–103.5)	51	101.5 (91.0–114.0)
Weight (kg)	20	71.4 (63.5–80.7)	52	74.7 (64.9–84.3)	52	77.5 (65.8–87.5)
BMI (kg/m^2^)	20	22.9 (21.4–24.6)	51	25.9 (22.7–27.9)	52	26.4 (22.2–31.7)
Systolic blood pressure (mmHg)	19	124.0 (118.0–132.0)	52	146.5 (140.0–157.0)	35	146.0 (130.0–159.0)
Diastolic blood pressure (mmHg)	19	67.5 (61.0–71.7)	52	79.0 (74.0–85.0)	35	75.0 (65.0–81.0)
Inflammation						
suPAR (ng/mL)	20	2.0 (1.8–2.2)	52	2.6 (2.2–3.1)	52	3.3 (2.6–4.7)
GDF15 (pg/mL)	5	199.1 (185.5–292.0)	52	1003.9 (829.4–1297.0)	52	1562.5 (1043.8–2186.2)
IL‐6 (pg/mL)	20	0.3 (0.3–0.3)	52	0.6 (0.3–0.9)	52	0.8 (0.6–1.6)
TNF‐α (pg/mL)	20	5.3 (5.1–5.7)	52	8.2 (6.4–9.8)	52	9.4 (7.3–14.0)
IL‐18 (pg/mL)	20	192.7 (154.0–235.3)	52	216.9 (167.0–344.4)	52	304.0 (197.7–404.1)
Biochemistry						
Albumin (g/L)	20	44.0 (40.5–45.0)	51	38.0 (36.0–40.0)	52	35.0 (32.0–38.0)
Alkaline phosphatase (U/L)	20	56.5 (48.5–74.5)	51	70.0 (58.0–80.0)	52	70.0 (56.0–85.0)
Bilirubin (μmol/L)	20	9.0 (6.0–11.0)	50	8.5 (6.0–11.0)	52	7.0 (6.0–9.5)
Blood urea nitrogen (mmol/L)	20	4.3 (3.7–5.1)	51	6.0 (5.4–6.7)	52	6.4 (5.2–8.5)
Creatinine (μmol/L)	20	82.0 (68.0–87.0)	51	81.0 (71.0–91.0)	52	86.5 (74.5–109.0)
CRP (mg/L)	20	0.4 (0.3–1.2)	51	1.2 (0.5–2.4)	52	3.2 (1.3–8.7)
Cystatin C (mg/L)	20	0.8 (0.8–0.9)	52	1.1 (0.9–1.2)	51	1.2 (1.1–1.6)
Hemoglobin (mmol/L)	20	8.9 (8.1–9.1)	51	8.7 (8.2–9.1)	51	8.0 (7.5–8.6)
Total cholesterol (mmol/L)	20	4.2 (3.8–4.3)	51	5.4 (4.6–6.2)	51	4.5 (4.0–5.5)
HDL (mmol/L)	20	1.6 (1.5–1.8)	51	1.6 (1.4–1.9)	51	1.4 (1.1–1.8)
LDL (mmol/L)	20	1.9 (1.7–2.2)	50	3.1 (2.4–3.8)	50	2.4 (1.7–3.1)
Triglycerides (mmol/L)	20	1.1 (0.9–1.6)	51	1.3 (1.0–1.9)	51	1.5 (1.0–1.9)
HbA1C (mmol/mol)	20	32.0 (28.5–33.0)	52	37.0 (36.0–39.0)	49	41.0 (38.0–45.0)
Frailty						
FI‐OutRef	20	1.0 (0.0–1.0)	50	1.0 (0.0–2.0)	52	3.2 (1.1–5.3)
Physical function						
4‐m gait speed (m/s)	20	1.4 (1.3–1.5)	52	1.3 (1.1–1.3)	50	0.8 (0.6–1.0)
Handgrip strength (kg)	20	44.0 (34.2–50.4)	52	30.8 (22.3–39.7)	51	25.4 (18.9–37.2)
Chair stand test (repetitions)	20	24.0 (21.0–27.5)	52	13.0 (11.0–16.0)	41	12.0 (9.0–14.0)
Cognitive function						
Trail making test A (seconds)	20	16.5 (13.7–19.4)	51	37.8 (29.0–54.6)	47	50.0 (37.0–69.0)
Trail making test B (seconds)	20	44.9 (36.6–53.3)	49	86.9 (67.0–113.4)	46	149.9 (101.0–246.0)
SDMT	20	60.0 (50.0–73.0)	50	37.0 (28.0–45.0)	46	25.2 (18.0–36.0)
HVLT‐R Total recall	20	31.0 (29.0–32.0)	52	24.5 (20.0–28.5)	40	20.0 (16.0–24.5)
Mortality						
Died	20	0 (0.0%)	52	4 (7.7%)	52	29 (55.8%)

*Note:* Data are reported as median (IQR) or *n* (%).

Abbreviations: BMI, body mass index; CRP, C‐reactive protein; FI‐OutRef, frailty index OutRef; GDF15, growth differentiation factor 15; HbA1c, glycated hemoglobin; HDL, high‐density lipoprotein; HVLT‐R, Hopkins Verbal Learning Test‐Revised; IL, interleukin; LDL, low‐density lipoprotein; SDMT, symbol digit modalities test; suPAR, soluble urokinase plasminogen activator receptor; TNF‐α, tumor necrosis factor‐α.

Six of the biomarkers included in the Olink Explore HT panel had previously been measured in the cohort using validated ELISA or Luminex assays. Overall, Olink results showed positive correlations with these immunoassays (Table S1), ranging from moderate correlations for tumor necrosis factor‐α (TNF‐α; *r* = 0.52) to strong correlations for soluble urokinase plasminogen activator receptor (suPAR; *r* = 0.88) and cystatin C (*r* = 0.88).

### 3.2 Identification of Proteins Associated With Biological Aging

**FIGURE 2:**
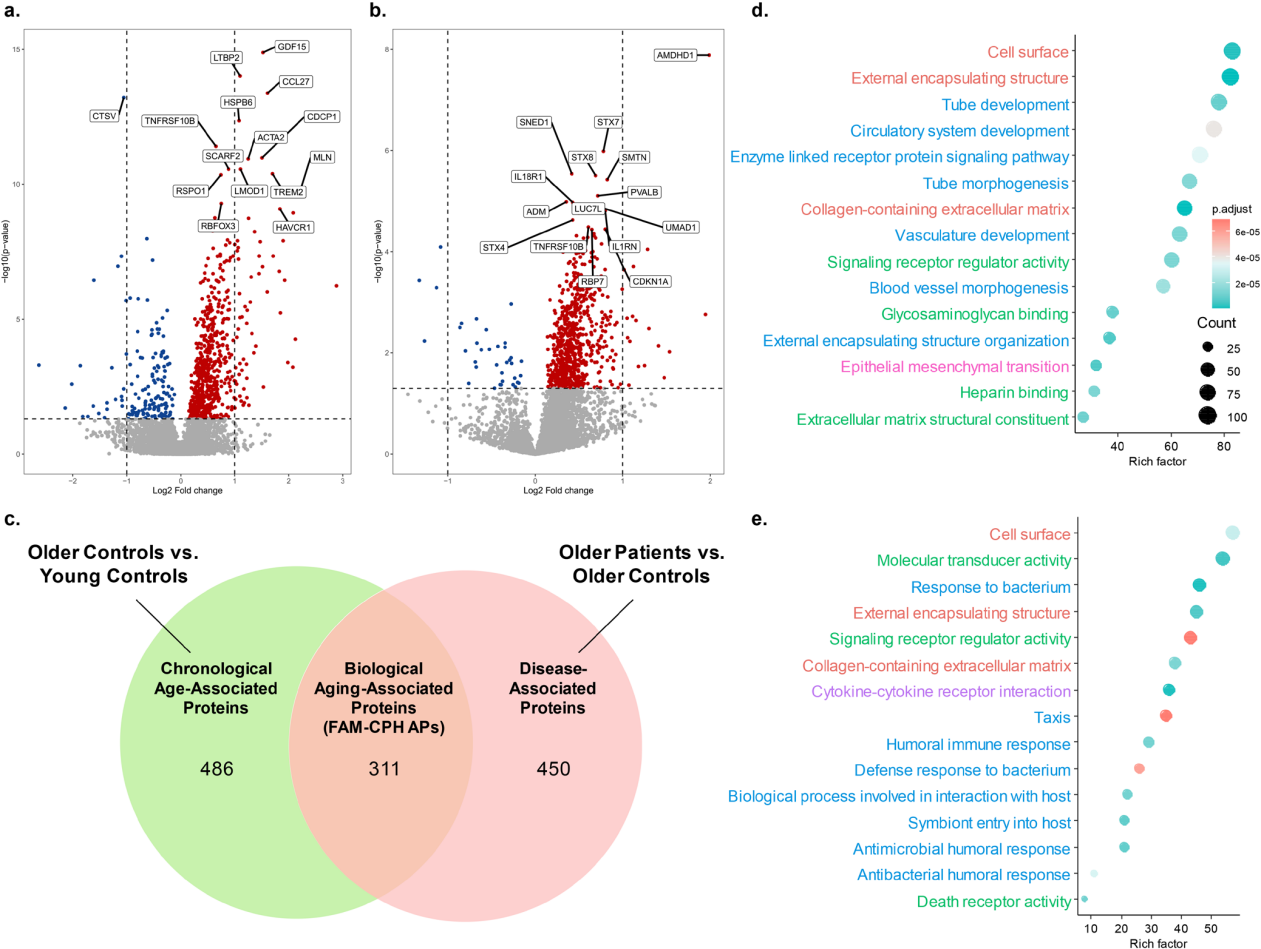
Plasma proteomic analysis identifies a protein signature associated with biological aging. (a) Volcano plots of differentially expressed proteins between Older Controls and Young Controls, representing proteins associated with age groups, and (b) between Older Patients and age‐matched Older Controls, representing proteins associated with disease burden. *p* Values estimated using *t*‐tests or Wilcoxon tests for matched (left) and unmatched (right) samples, depending on normality assumption, and were corrected for multiple comparisons using the Benjamini‐Hochberg method. The *p* values are plotted as −log10(*p*‐value) on the vertical axis. The horizontal dotted line represents *p* values equal to 0.05. On the horizontal axis, log2 fold changes are plotted. The vertical dotted lines mark a log2 fold change of −1 and 1, which corresponds to a halving or doubling in the protein expression. Red dots indicate significantly upregulated proteins, blue dots indicate significantly downregulated proteins, and gray dots indicate proteins with no significant difference in expression levels. Top 15 significant *p* values (*p* < 0.05) are labeled with the abbreviated names of the corresponding protein. (c) Venn diagram depicting the overlap of differentially expressed proteins (*p* < 0.05) between the analyzed groups, representing “Chronological Age‐Associated Proteins”, “Disease‐Associated Proteins”, and FAM‐CPH APs. (d) Functional enrichment analyses of 486 Chronological Age‐Associated Proteins, and (e) 311 FAM‐CPH APs. Databases used: GO (biological processes: Blue; molecular function: Green; cellular component: Red), KEGG (purple), Reactome (orange), Hallmarks (pink), using hypergeometric test. Rich factor represents the ratio of observed to expected proteins in each pathway, indicating the degree of enrichment; a higher value reflects stronger enrichment. The *p* values were corrected for multiple testing using the Benjamini‐Hochberg method. Dot sizes represent the number of proteins, and dot colors represent significance levels.

To identify proteins associated with chronological age groups and disease burden, we conducted pairwise differential expression analyses between participant groups. Comparison of Older Controls to Young Controls identified 797 Chronological and Biological Aging‐Associated Proteins, with 635 proteins being upregulated and 162 downregulated with older age (Figure 2a and Table S2). Similarly, comparison of Older Patients and Older Controls identified 761 Disease and Biological Aging‐Associated Proteins, with 724 proteins upregulated and 37 downregulated with increased disease burden (Figure 2b and Table S2). Median expression levels of the top 20 differentially expressed proteins from each comparison are shown in Figures S2 and S3.

To pinpoint potential candidate biomarkers of biological aging, we focused on the overlap between Chronological Age‐Associated proteins and Disease‐Associated Proteins (Figure 2c). At this intersection, we identified 311 proteins whose expression differed both between Young and Older Controls and between Older Controls and Older Patients, from here‐on referred to as FAM‐CPH biological Aging Proteins (FAM‐CPH APs), while 486 exclusively Chronological Age‐Associated Proteins and 450 exclusively Disease‐Associated Proteins remained. FAM‐CPH APs included a similar proportion of Chronological and Biological Aging‐Associated Proteins (39.0%) and Disease and Biological Aging‐Associated Proteins (40.9%). Among FAM‐CPH APs, 302 were upregulated and 9 downregulated with older age, while 292 were upregulated and 19 downregulated with increased disease burden.

To determine whether the groups of 486 Chronological Age‐, 450 Disease‐Associated Proteins, and 311 FAM‐CPH APs identified from the different pairwise comparisons reflected distinct biological processes, we performed functional enrichment analyses using GO, KEGG, Reactome, and Hallmarks databases. The 486 proteins differentially expressed exclusively between Older Controls and Young Controls—reflecting Chronological Age‐Associated Proteins—were enriched in pathways related to extracellular matrix organization, vasculature development, and cell signaling (Figure 2d), whereas the 450 Disease‐Associated Proteins differentially expressed exclusively between Older Patients and Older Controls were not enriched for any of the tested pathways. Enrichment analysis of the 311 FAM‐CPH APs revealed involvement of pathways related to immune function and host‐pathogen interactions. Both Chronological Age‐Associated proteins and FAM‐CPH APs were enriched for components of the extracellular matrix and of the cell surface (Figure 2e).

### 3.3 Comparisons With Published Proteomic Signatures of Aging, Disease, and Senescence

To contextualize and validate our findings, we compared the three identified protein categories: 486 Chronological Age‐Associated Proteins, 450 Disease‐Associated Proteins, and 311 FAM‐CPH APs, with proteins previously reported to be associated with chronological age, disease, and geriatric syndromes such as frailty, or proteins secreted as part of the SASP. This was done through a targeted PubMed literature search (see Section 2). Across 35 targeted proteomic studies in human plasma or serum, we compiled lists of known age‐ (19 studies) or disease‐associated proteins (16 studies). We also compiled a list of known SASP‐associated proteins (12 studies). The full lists can be found in Tables S3–S5.

#### 3.3.1 Proteomic Signatures Associated With Age

Multiple studies have investigated the relationship between chronological age and protein expression in generally healthy populations and community‐based cohorts. From 19 published studies (see Section 2), we compiled a list of 5285 known age‐associated proteins (Table S3a), of which 3148 (59.7%) were included in the Olink HT panel. Several of these studies also proposed reduced protein signatures most strongly associated with age, which are listed in Table S3b. At the individual study level, the overlap between known age‐associated proteins and FAM‐CPH APs ranged from 10.2% to 36.7% (Table S3c). Together, known age‐associated proteins accounted for 84.4% (410 proteins) of the Chronological Age‐Associated Proteins, 78.4% (353 proteins) of the Disease‐Associated Proteins, and 92.3% (287 proteins) of the FAM‐CPH APs, suggesting that while our FAM‐CPH APs significantly overlap with known age‐associated proteins, they also contain 24 potentially novel markers not previously linked to chronological age in the literature.

#### 3.3.2 Proteomic Signatures Associated With Disease

As aging is a major driver of most age‐related diseases and mortality risk, we examined whether our three categories of proteins had previously been identified in proteomic studies of disease and frailty. From 16 published studies (see Section 2), we identified 2170 known disease‐associated proteins (Table S4a), of which 1782 (82.1%) were present in the Olink HT panel. Several of these studies also proposed reduced protein signatures most strongly associated with disease, which are listed in Table S4b. At the individual study level, the overlap between known disease‐associated proteins and FAM‐CPH APs ranged from 0.0% to 47.0% (Table S4c). Together, known disease‐associated proteins accounted for 66.5% (323 proteins) of the Chronological Age‐Associated Proteins, 52.9% (238 proteins) of the Disease‐Associated Proteins, and 85.9% (267 proteins) of the FAM‐CPH APs, suggesting that FAM‐CPH APs also contain proteins linked to disease processes.

#### 3.3.3 Proteomic Signatures Associated With the SASP

We next evaluated the overlap with known SASP‐associated proteins based on 12 studies identifying proteins as being upregulated by senescent human cells and released as part of the SASP (see Section 2). A total of 5696 known secreted SASP proteins were identified in the literature (Table S5), of which 1876 (32.9%) were present in the Olink HT panel. Together, these known SASP proteins accounted for 44.4% (216 proteins) of the Chronological Age‐Associated Proteins, 47.8% (215 proteins) of the Disease‐Associated Proteins, and 46.3% (144 proteins) of the FAM‐CPH APs, indicating a potential contribution of cellular senescence to biological aging.

#### 3.3.4 Combined Proteomic Signatures of Age, Disease, and SASP

**FIGURE 3:**
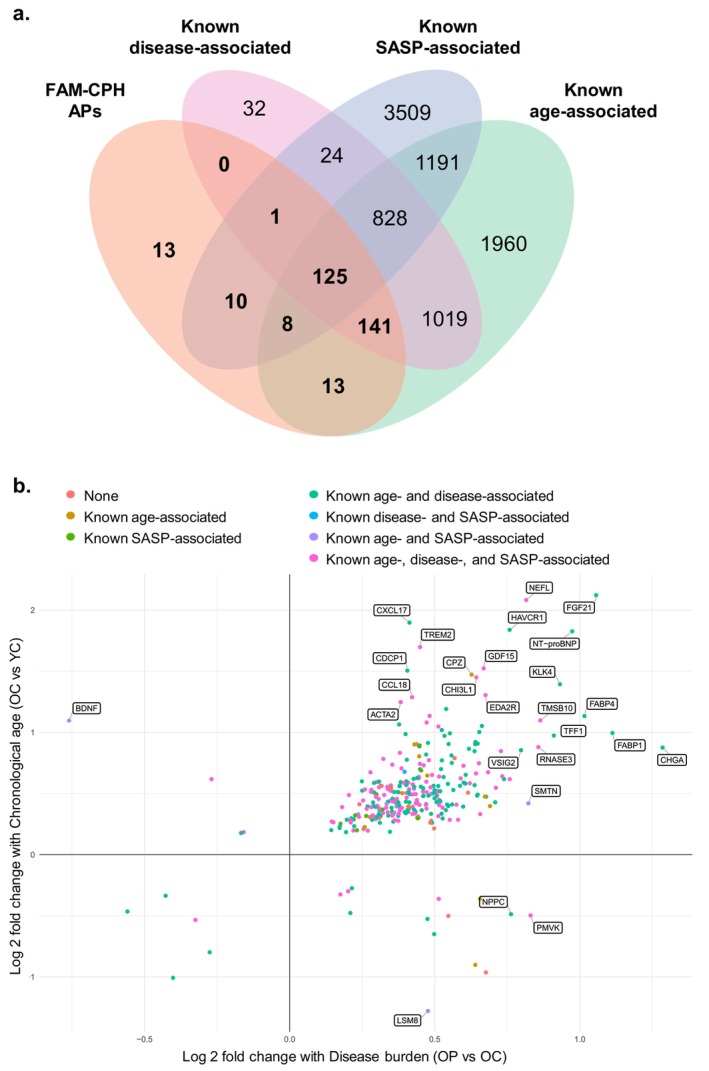
FAM‐CPH APs show substantial overlap with known age‐, disease‐, and SASP‐associated proteins. (a) Venn diagram depicting the overlap between FAM‐CPH APs (in bold) and known age‐, disease‐, and SASP‐associated proteins previously identified in the proteomics literature. (b) Scatter plot comparing log2‐fold changes of FAM‐CPH APs with disease burden (horizontal axis; OP vs. OC), and with chronological age (vertical axis; OC vs. YC). Each dot represents a protein, and each color indicates whether the protein has previously been identified as an age‐, disease‐, or SASP‐associated protein, or any combination thereof. The 15 proteins with the largest absolute fold change on either axis are labeled with the corresponding abbreviated protein names. OC, Older Controls; OP, Older Patients; YC, Young Controls.

We then combined the PubMed‐derived lists of known age‐, disease‐, and SASP‐associated proteins, yielding 8861 unique proteins, of which 3706 (41.8%) were included in the Olink HT panel. Among our identified proteins, 434 (89.3%) Chronological Age‐Associated Proteins, 399 (88.7%) Disease‐Associated Proteins, and 298 (95.8%) FAM‐CPH APs overlapped with the literature‐based list. These results support the ability of our study design to detect proteins associated with biological aging and reflecting both age‐ and disease‐related processes, such as GDF15 and PLAUR (suPAR), TNFRSF1A, TNFRSF1B, cystatin C, HAVCR1, SPON1, EDA2R, and IGFBP4. Notably, several proteins frequently reported as age‐associated in the PubMed literature, such as the inflammatory biomarkers TNF‐α and IL‐6, MMP12, FAS, EGFR, and SOST were identified as Chronological Age‐Associated Proteins, and others, such as PTN and STC1 as Disease‐Associated Proteins, but none of these were part of the FAM‐CPH APs. Importantly, 13 FAM‐CPH APs had not been previously associated with chronological age, disease, or the SASP in proteomic studies, highlighting a set of potentially novel biomarkers uniquely identified through our approach (Figure 3). A detailed list of these proteins can be found in Table S6.

### 3.4 Unsupervised Clustering Identifies Protein Expression Patterns Across Age and Disease

**FIGURE 4:**
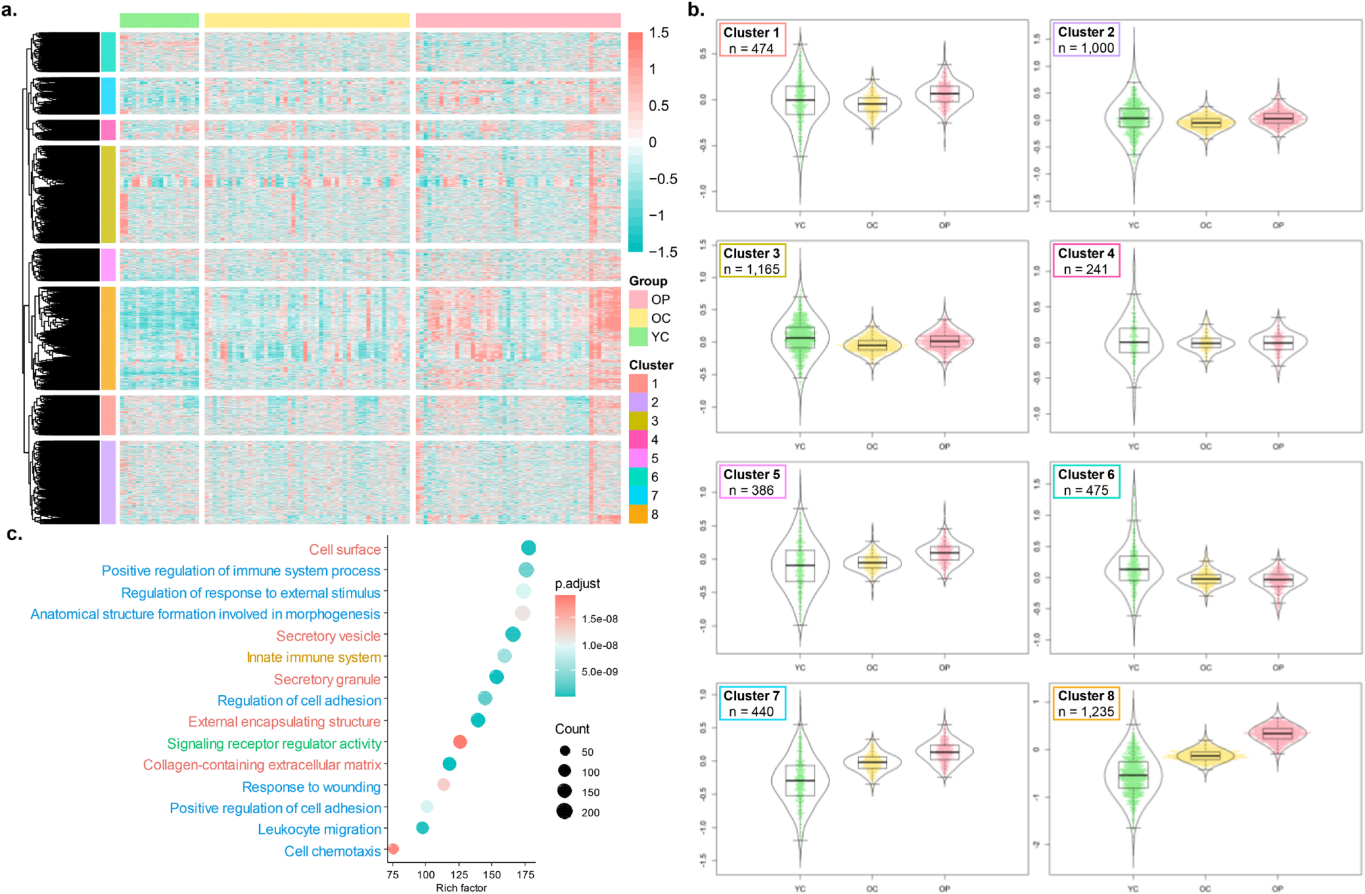
Unsupervised hierarchical clustering identifies a cluster enriched in FAM‐CPH APs. (a) Dendrogram and heatmap of identified clusters following unsupervised hierarchical clustering analyses of all 5416 plasma proteins based on expression levels across all study participants (*n* = 124). Matched Older Control‐Older Patient pairs are shown in the same order. Participant groups are labeled as YC (Young Controls), OC (Older Controls), and OP (Older Patients). The color bars next to the dendrogram denote the individual clusters. The heatmap indicates the expression profile for each protein for each participant, stratified by participant groups. The red color indicates a relative higher expression and the blue color a relative lower expression of the protein compared to the mean expression level across all study participants. (b) Boxplots, dot plots, and violin plots showing mean relative expression of the proteins in each cluster by participant group. Horizontal lines and boxes indicate median and interquartile ranges (IQRs), and whiskers extend to 1.5 times the IQR. (c) Functional enrichment analysis of Cluster 8. Databases used: GO (biological processes: Blue; molecular function: Green; cellular component: Red), KEGG (purple), Reactome (orange), Hallmarks (pink), using hypergeometric test. The *p* values were corrected for multiple testing using the Benjamini‐Hochberg method. Dot size represents the number of proteins, and dot colors represent significance levels.

To complement and validate the group‐based analyses, we next used an unbiased data‐driven approach to assess whether the FAM‐CPH AP signature could be recapitulated when examining broader patterns of coordinated protein expression across all study participants. Therefore, we performed unsupervised hierarchical clustering on all 5416 proteins across all three participant groups (*n* = 124), using complete linkage and Euclidean distance as the dissimilarity measure. This analysis identified 8 distinct protein clusters ranging in size from 241 (Cluster 4) to 1235 (Cluster 8) proteins (Figure 4a and Table S7). Several clusters (5, 7, and 8) were characterized by progressive protein upregulation from Young Controls to Older Controls, and further to Older Patients, whereas Cluster 6 showed an opposite trend (Figure 4b).

We then evaluated each cluster for overrepresentation of the protein categories identified in the group‐based analyses. Chronological Age‐associated Proteins were distributed across multiple clusters, including 10.3% in cluster 5 (50/486 proteins, Benjamini‐Hochberg‐adjusted *p* = 0.01), 14.2% in cluster 7 (69/486, *p* = 4.16 × 10^−6^), and 42.2% in cluster 8 (205/486, *p* = 3.13 × 10^−23^). Disease‐Associated Proteins were mainly contained in Cluster 8 (286/450 proteins, 63.6%, *p* = 2.23 × 10^−84^). Cluster 8 also contained the vast majority of FAM‐CPH APs with 264 of the 311 proteins in this group (84.9%, *p* = 1.11 × 10^−128^), providing supporting evidence for the FAM‐CPH AP signature identified in the group‐based analyses. Functional enrichment analysis of Cluster 8 proteins highlights pathways related to innate immunity, inflammation, and the regulation of cell migration and adhesion (Figure 4c).

To assess the robustness of the clusters, we conducted a sensitivity analysis using Manhattan distance as the dissimilarity measure. This method yielded 7 clusters, including one cluster significantly enriched with FAM‐CPH‐APs. Consistent with the Euclidean‐based results, this cluster also contained 264 FAM‐CPH APs, with 244 overlapping proteins between the two methods, underscoring the stability of our clustering results.

### 3.5 Identification of a Protein Signature Associated With Mortality Risk

To identify a protein signature associated with increased mortality risk, we conducted an exploratory analysis using a regularized Cox regression with a LASSO penalty to analyze the association between the 311 FAM‐CPH APs and time‐to‐death. The model included unpenalized adjustment for sex and baseline hazard stratified by group. Young Controls were excluded from this analysis as there were no deaths during follow‐up in this group. Among older participants (*n* = 104), we identified a signature of 6 proteins most strongly associated with mortality through cross‐validation (NPC2, GDF15, FBLN2, LZTS1, KLK4, and ACTA2; Figure S7). Stability assessment of the 6 selected FAM‐CPH APs via stratified bootstrapping revealed 2 proteins consistently selected across all iterations, NPC intracellular cholesterol transporter 2 (NPC2) and kallikrein‐related peptidase 4 (KLK4), highlighting these as robust candidates for future validation.

**FIGURE 5:**
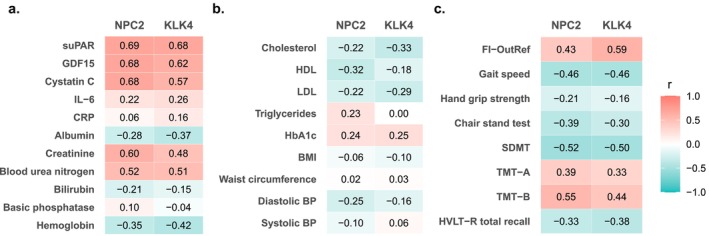
The two proteins robustly associated with mortality risk are correlated with aging‐related measures. Pearson correlation coefficients (*r*) are shown for pairwise correlations between each protein and (a) inflammation and routine biochemistry. (b) metabolic and clinical measures. (c) measures of frailty, physical and cognitive function. BMI, body mass index; BP, blood pressure; CRP, C‐reactive protein; FI‐OutRef, frailty index OutRef; GDF15, growth differentiation factor 15; HDL, high‐density lipoprotein; HVLT‐R, Hopkins verbal learning test; IL‐6, interleukin‐6; LDL, low‐density lipoprotein; SDMT, symbol digit modalities test; suPAR, soluble urokinase plasminogen activator receptor; TMT, trail making test.

We evaluated correlations between expression levels of NPC2 and KLK4 and aging‐related measures, including inflammation and organ function biomarkers, metabolic and clinical markers, as well as physical and cognitive function. Both proteins showed strong correlations with systemic chronic inflammation biomarker suPAR and with GDF15 and cystatin C (ranging from *r* = 0.57 to *r* = 0.69, Figure 5 and Table S8), whereas correlations with acute inflammation markers CRP and IL‐6 were weaker (*r* = 0.06 to *r* = 0.26). Additionally, both proteins correlated strongly with blood urea nitrogen, creatinine, the frailty index FI‐OutRef (*r* = 0.43 to *r* = 0.60), and with lower gait speed and cognitive function (*r* = −0.46 to *r* = 0.55). Correlations with metabolic measures were generally weak (*r* = −0.33 to *r* = 0.25).

## 4 Discussion

In this study, we performed a large‐scale proteomic analysis using the Olink Explore HT platform to investigate plasma protein profiles associated with chronological age groups, disease burden, and at their intersection, biological aging. We used a unique design comparing three distinct groups: older patients with unselected conditions, age‐matched older individuals with a low disease burden, and healthy young individuals, and identified 311 FAM‐CPH APs that were differentially expressed in both age‐related and disease‐related comparisons, suggesting potential relevance as biomarkers of biological aging. While many of these proteins had previously been associated with chronological age, disease or geriatric syndromes, supporting the validity of our approach, a subset of proteins had not been previously reported in the context of aging or disease proteomes, representing novel candidates for further investigation. Unsupervised hierarchical clustering revealed that most of the 311 biological aging‐associated proteins co‐occurred within a single cluster, suggesting potential involvement in shared pathways linked to both aging and disease. Finally, we identified a reduced two‐protein signature robustly associated with mortality risk and correlated with aging‐related measures.

A key strength of this study lies in the inclusion of a clinically heterogeneous older population, including both patients with multimorbidity and age‐matched healthier individuals, in contrast to previous aging proteomic studies, which have primarily focused on generally healthy or tightly controlled cohorts (Lehallier et al. 2019; Tanaka et al. 2018). While controlling for specific backgrounds or diseases may reduce variability, it also limits generalizability to real‐world aging populations, where multimorbidity and physiological complexity are the norm rather than the exception (Barnett et al. 2012; Pati et al. 2023). By distinguishing between populations on different aging trajectories, our design further captures the complexity of aging and allows for the identification of proteins that are relevant to biological aging beyond those associated with chronological age alone, providing a more representative view of aging‐related processes. The matched control group increases the likelihood that observed differences are biologically relevant rather than driven by confounding demographic factors. Our study design, therefore, provides a robust framework to dissect the complex interplay between chronological age and disease effects on plasma protein expression.

We identified 797 proteins differentially expressed between chronological age groups, 761 differentially expressed between disease burden groups, and a subset of 311 proteins overlapping between these two groups. Consistent with previous large proteomic studies, which on average report 75% of proteins to be upregulated and 25% downregulated with age, we also found that the majority of proteins were upregulated in the older age group (80%) and in patients with an increased disease burden (95%) (Coenen et al. 2023; Goeminne et al. 2025; Lehallier et al. 2019; Lind et al. 2019; Moaddel et al. 2025; Oh et al. 2023; Sathyan, Ayers, Gao, Weiss, et al. 2020; Tanaka et al. 2020; Tin et al. 2019). This pattern may reflect the activation of biological processes associated with aging, such as chronic immune activation, chronic inflammation, and impaired proteostasis. The particularly low proportion of down‐regulated proteins with age among the 311 FAM‐CPH APs (3%) may further reflect a selection of proteins that are also associated with disease burden, which capture additional cellular stress and dysregulation and can be expected to be more frequently upregulated with age. FAM‐CPH APs contained several well‐established age‐associated proteins, including GDF15, PLAUR (suPAR), HAVCR1, SPON1, CHI3L1, CST3, ADM, and proteins from the IGF‐family (IGFBP4 and 7) (Argentieri et al. 2024; Coenen et al. 2023; Goeminne et al. 2025; Lehallier et al. 2019; Moaddel et al. 2025; Mörseburg et al. 2025; Oh et al. 2025, 2023; Sathyan, Ayers, Gao, Weiss, et al. 2020; Tanaka et al. 2020, 2018; Wang, Huang, et al. 2025). Many of these proteins also ranked among the top biomarkers in proteomic studies predicting disease incidence and frailty (Carrasco‐Zanini et al. 2024; Gadd et al. 2024; Kuo et al. 2024, 2025; Liu et al. 2022; Sathyan, Ayers, Gao, Milman, et al. 2020; Sathyan et al. 2025; Ubaida‐Mohien et al. 2025). The consistent reporting of these proteins in previous plasma or serum proteomic studies and in the present study, despite differences in sample size, population age, and health status, supports the robustness of our approach in identifying proteins reflecting biological aging processes at the intersection of aging and disease. Furthermore, these hypothesis‐driven findings from group comparisons were broadly reproduced in the unsupervised data‐driven clustering analysis, further supporting their validity. A strength of this analysis is the clear and transparent criteria used to select the number of clusters, complemented by a stability assessment to enhance the robustness of the results. This assessment evaluates whether applying the clustering method to multiple datasets from the same data‐generating process would yield consistent clusters, a criterion that should be desirable when performing exploratory data analysis (von Luxburg 2010). Despite this, such stability assessments, as well as detailed reporting on cluster selection or hyperparameter tuning in penalized regression methods (and the resulting protein signatures), are often overlooked, raising concerns about the generalizability of reported findings.

Leveraging one of the largest protein coverages to date (5416 unique proteins), we identified 13 candidate novel biomarkers of biological aging not previously reported in selected proteomic studies. While some may have been described in other age‐ or disease‐related contexts, their identification in this study expands the current repertoire of aging‐associated biomarkers. These proteins warrant further investigation to elucidate their potential involvement in aging mechanisms. Notably, several appear to be involved in immune‐related processes (e.g., RC3H1), cell cycle regulation (e.g., LZTS1), or protein homeostasis (e.g., ZER1 and ZFAND2B), suggesting potential relevance to key biological pathways in aging. It is important to note, however, that despite its broad coverage, the overlap between the Olink HT platform and protein lists reported in the literature derived from other high‐throughput platforms such as SomaScan and other Olink panels was estimated to be around 65%. For example, known age‐ and disease‐related proteins such as ADAMTS5, KLK3, LTBP4, NTN1, or PLAT are not included in Olink HT. This incomplete overlap not only prevents us from integrating aging clocks previously defined using Olink Explore 3072 or 1536 into our study, but also indicates that additional candidate biomarkers could be detected using other platforms on study designs similar to ours. Of note, several well‐known age‐ or disease‐associated proteins, such as IL‐6, TNF‐α, PTN, MMP12, EGFR, SOST, or RET (Argentieri et al. 2024; Carrasco‐Zanini et al. 2024; Coenen et al. 2023; Gadd et al. 2024; Lehallier et al. 2019; Tanaka et al. 2018), were not categorized as biological aging‐associated proteins in our analysis. While most of these were elevated in Older Controls compared to Young Controls, they did not show differential expression with disease burden; except for PTN, which was differentially expressed with disease burden but not with age. The absence of such well‐established age‐associated proteins among FAM‐CPH APs may be attributed to several factors, including study design, statistical power, cohort characteristics, and technical differences between proteomic platforms, rather than indicating a lack of relevance to the biological aging process. Whereas most previous proteomic aging studies have analyzed individuals spanning a wide age range and modeled age as a continuous variable (Coenen et al. 2023; Lehallier et al. 2019; Moaddel et al. 2025; Tanaka et al. 2020, 2018), our analysis compared two distinct age groups. As a result, our approach is more likely to detect proteins exhibiting pronounced changes between young and older adults, and while our analysis does not assume linearity of these changes between the two age categories, proteins that change gradually or transiently across the lifespan, particularly during mid‐ and late life, may not be captured by this design. The limited statistical power of our study may further reduce our ability to capture true biological differences, and biological heterogeneity, such as genetic background and population‐specific factors (including age distribution and disease burden), may also contribute to the observed discrepancies between our findings and those of other studies. Possibly reflecting methodological influences, we observed that proteins with lower correlations between Luminex and Olink's PEA assay, such as IL‐6 and TNF‐α, were differentially expressed in both age and disease burden comparisons when measured using Luminex, but only in age comparisons when measured using Olink's PEA assay. In contrast, proteins with stronger inter‐assay correlations, such as suPAR and GDF15, were differentially expressed with both age and disease burden when measured using Luminex or Olink's PEA assay.

Nevertheless, the substantial overlap between key proteins associated with both age and disease aligns with the geroscience hypothesis that shared mechanisms underlie aging and disease processes. However, identifying mechanistic biomarkers of biological aging requires more than statistical association; it also necessitates demonstration of functional involvement in the biological pathways driving aging and disease. Some of the proteins frequently identified in proteomic studies, such as the inflammation marker suPAR—the soluble form of the uPAR receptor—have been mechanistically implicated in renal and cardiovascular disease (Hayek et al. 2015; Hindy et al. 2022; Rasmussen, Petersen, and Eugen‐Olsen 2021), while the causal involvement of other proteins in aging and disease remains unclear. In addition, it will be important to distinguish proteins that actively contribute to cellular dysfunction and pathology from those that reflect compensatory or adaptive responses to physiological stress. Elucidating these functional roles will require targeted mechanistic studies, including experimental models and longitudinal studies in humans. As observed in previous studies (Tanaka et al. 2018), nearly half of FAM‐CPH APs have previously been characterized as SASP proteins, providing insight into the shared mechanisms linking aging and disease, such as cellular senescence. Additionally, unsupervised hierarchical clustering analysis revealed that many of the FAM‐CPH APs followed similar expression patterns across the three participant groups, suggesting that these proteins may be co‐regulated or involved in related biological pathways. Functional enrichment analysis of FAM‐CPH APs revealed enrichment for proteins related to extracellular matrix organization, vasculature, and cell signaling, consistent with recent proteomic studies in which age‐associated proteins map to similar biological pathways, in particular those related to extracellular matrix organization (Coenen et al. 2023; Kuo et al. 2025; Lehallier et al. 2019; Moaddel et al. 2025; Sathyan, Ayers, Gao, Weiss, et al. 2020; Tanaka et al. 2020, 2018). Similarly, we observed enrichment of pathways related to immune function, inflammatory signaling, and the regulation of cell adhesion among proteins in Cluster 8, in line with previous studies (Goeminne et al. 2025; Kuo et al. 2025; Moaddel et al. 2025; Sathyan, Ayers, Gao, Weiss, et al. 2020; Tanaka et al. 2020). Altogether, these pathways are evocative of hallmarks of aging such as inflammaging, immunosenescence, and cellular senescence.

Although large‐scale plasma proteomic studies have allowed the identification of thousands of unique proteins associated with age or disease, illustrating the physiological complexity of biological aging, there is a need to develop more targeted, interpretable, and actionable proteomic signatures to facilitate the clinical translation of these biomarker discoveries. The use of multiple biomarkers or biomarker panels, rather than single biomarkers, is better suited to capture the complexity of the aging process. Several studies have developed reduced proteomic signatures to predict chronological age, disease, frailty, or mortality using different approaches such as elastic net or LASSO regressions, or gradient boosting machine learning models (Argentieri et al. 2024; Coenen et al. 2023; Kuo et al. 2024; Lehallier et al. 2019; Sathyan, Ayers, Gao, Weiss, et al. 2020; Tanaka et al. 2020, 2018). Differences in study designs, objectives, and methodological approaches have led to variation in the composition and size of reduced proteomic signatures, ranging from a single protein to several hundreds. Similarly, recommendations on blood‐based biomarkers to be used in geroscience trials have been proposed by the TAME (Targeting Aging with Metformin) Workgroup (Justice et al. 2018); out of 10 recommended proteins, 7 are included in Olink HT, and of these, 5 (TNFRI and II, GDF15, cystatin C, and NT‐proBNP) were among the 311 FAM‐CPH APs. While a larger sample might have allowed identification of a broader panel, we were able to identify a robust two‐protein signature (KLK4 and NPC2) associated with mortality risk using Cox regression with LASSO penalty. Both proteins have previously been associated with age and disease in proteomic studies (Argentieri et al. 2024; Basisty et al. 2020; Carrasco‐Zanini et al. 2024; Gadd et al. 2024; Kuo et al. 2024; Lehallier et al. 2019; Sun et al. 2023; Tin et al. 2019; Xu et al. 2025). NPC2 is involved in lysosomal function and is expressed on the surface of senescent cells (Deng et al. 2024), while KLK4 is a protease implicated in extracellular matrix degradation and has been linked to chronic kidney disease (Papagerakis et al. 2015; Wruck et al. 2022), supporting the validity of our findings. Additionally, in our study, both showed strong correlations with suPAR, a biomarker of chronic inflammation and biological aging, and a strong predictor of disease and mortality (Rasmussen, Caspi, et al. 2021; Rasmussen, Petersen, and Eugen‐Olsen 2021). Future studies to identify the most predictive, prognostic, and intervention‐responsive proteins among the 311 FAM‐CPH APs could employ a multi‐step approach, first assessing each protein's correlation with biological age and age acceleration measures, prioritizing proteins with strong associations across multiple aging biomarkers. Second, multivariate analyses should be conducted to identify proteins associated with age‐related outcomes, independent of traditional risk factors. Notably, several of the FAM‐CPH APs, including suPAR and GDF15, have previously been independently associated with chronic diseases, decline in physical and cognitive function, and increased mortality (Rasmussen, Caspi, et al. 2021; Rasmussen, Petersen, and Eugen‐Olsen 2021; Tavenier, Andersen, et al. 2021). Similar analyses should be made for novel and not previously identified targets. Third, the responsiveness of the proteins to geroprotective interventions should be evaluated. Finally, cross‐validation in independent cohorts and composite biomarker construction can help refine the panel to a feasible size while maintaining high predictive power for aging outcomes and intervention responses.

This study has limitations. First, the small sample size, particularly the limited number of young controls, may limit the detection of more subtle proteomic differences between the groups. This limitation may be especially relevant for down‐regulated proteins, as these may show smaller age‐associated changes. The small sample size also limited our ability to evaluate sex differences in age‐ and disease‐associated proteins in sex‐stratified analyses. Second, although the study design allows us to disentangle age‐related from disease‐related effects, the relatively large age gap between young and older participants may influence the findings and their interpretation, particularly in comparison to previous studies that have examined age effects on a continuous scale (Coenen et al. 2023; Lehallier et al. 2019; Tanaka et al. 2020, 2018). Thus, the cross‐sectional and categorical design may bias the findings toward the detection of proteins with larger age‐related changes while limiting the detection of small, gradual changes across the lifespan. Third, we lack information on the types and numbers of chronic disease diagnoses for the Older Patients population. While this population is likely highly heterogeneous, the overrepresentation of specific conditions may introduce bias. Fourth, the single‐site nature of the study may introduce bias through population‐specific influences on aging and disease. Fifth, on a technical level, moderate correlations have been reported between the Olink PEA and SomaScan platforms (Eldjarn et al. 2023; Rooney et al. 2025), which could introduce a bias in our results. However, our findings were supported by a high degree of overlap in age‐ and disease‐associated proteins with previous studies. Furthermore, we observed moderate to high correlations (*r* = 0.52–0.88) between Olink PEA and ELISA/Luminex‐based measurements for several proteins within our cohort. Furthermore, while our study focused on soluble circulating proteins, emerging studies of exosome‐derived proteins of aging and senescence may reveal additional relevant biomarkers (Patel et al. 2025).

Future studies integrating additional omics data, such as epigenomics, transcriptomics, or metabolomics, could provide deeper insights into the molecular mechanisms of biological aging and help identify biologically and clinically relevant biomarkers. In this context, various biological aging clocks based on omics data have been developed to act as outcome measures for the effectiveness of interventions targeting aging processes (Rutledge et al. 2022; Shen et al. 2024). Although technical challenges related to coverage, reproducibility, and platform variability remain, proteomics offers the advantage of greater accessibility and provides a more functionally relevant readout of biological state by directly measuring the abundance of proteins, the primary effectors of cellular function, and the ultimate targets of most therapeutic interventions.

In summary, we identified both established and novel biomarkers differing between older and young adults and between individuals with low and high disease burden as potential candidate biomarkers of biological aging. These findings provide insight into the shared molecular mechanisms underlying age‐ and disease‐related processes. These biomarkers may be valuable in future studies aimed at assessing biological age and evaluating the efficacy of therapeutic interventions targeting aging mechanisms. The overlap between age‐ and disease‐associated proteins supports the geroscience hypothesis, suggesting that interventions targeting fundamental aging mechanisms may benefit a wide range of chronic conditions. Moreover, the identification of previously unreported proteins highlights the value of studying clinically diverse populations and underscores the potential for discovering biomarkers with future utility for risk stratification, monitoring, and therapeutic development in aging‐related health care.

## Author Contributions

J.T., M.B.H., A.L.A., O.A., and L.J.H.R. conceptualized and designed the study. J.T., M.B.H., A.L.A., and L.J.H.R. contributed to data acquisition. J.T., L.J.H.R., N.N.H., and T.K. performed the analyses. J.T. and L.J.H.R. drafted the paper. All authors provided the critical input on the results, reviewed the manuscript, and approved the final version.

## Funding

This work was supported by Læge Sofus Carl Emil Friis og Hustru Olga Doris Friis’ Legat. Beckett‐Fonden, 23‐2‐11663.

## Ethics Statement

The study protocol was approved by the Health Research Ethics Committee for the Capital Region of Denmark (ref.# H‐16038786) and the Danish Data Protection Agency (ref.# AHH‐2016‐067) and registered on clinicaltrials.gov (NCT03052192). The study was conducted in accordance with the Declaration of Helsinki, and all participants gave written informed consent.

## Conflicts of Interest

The authors declare no conflicts of interest.

## Supporting information


**Figure S1:** Overview of analyses performed in the study.
**Figure S2:** Boxplots, dot plots, and violin plots for proteins with the highest differential expression between Older Controls and Young Controls representing “Chronological Age‐Associated Proteins”. Plots show median and interquartile range for each group: Young Controls (YC, green), Older Controls (OC, yellow), and Older Patients (OP, red). Boxplots display median and interquartile range, and whiskers extending to 1.5 times the IQR.
**Figure S3:** Boxplots, dot plots, and violin plots for the 20 proteins with the highest differential expression between Older Patients and age‐matched Older Controls representing Disease‐Associated Proteins. Plots show median and interquartile range for each group: Young Controls (YC, green), Older Controls (OC, yellow), and Older Patients (OP, red). Boxplots display median and interquartile range, and whiskers extending to 1.5 times the IQR.
**Figure S4:** Scree plot of dendrogram agglomerations for the main cluster analysis presented in the paper using Euclidean distance as dissimilarity measure.
**Figure S5:** Normalized stability score of the Euclidean distance‐based hierarchical clustering with complete linkage by number of clusters based on the Rand index for sampling proportion 𝜈 = 0.95, 0.90, and 0.85. The normalized stability score was estimated using normalization by random labels.
**Figure S6:** Scree plot of dendrogram agglomerations for the cluster analysis using Manhattan distance as dissimilarity measure.
**Figure S7:** Stability assessment of protein selection procedure based on regularized Cox regression with LASSO penalty. The entire selection procedure was repeated across 1000 stratified bootstrap samples. The proteins presented were nonzero within a 1‐standard‐error range of the optimal model with the lowest partial likelihood deviance based on the full dataset. The blue color indicates nonzero proteins selected in the optimal model. Left: Boxplots of the 1000 bootstrap realizations of the coefficients of the presented proteins. Right: Proportion of times the coefficient is zero in the bootstrap distribution.

**Table S1:** Correlations between NPX values from Olink Explore HT and immunoassay measurements for selected biomarkers.
**Table S2:** Median NPX (IQR) value by group and fold changes and *p* values for each comparison.
**Table S3a:**. Combined list of age‐associated proteins from the literature.
**Table S3b:**. Selected panels of age‐associated proteins from the literature.
**Table S3c:** Individual study comparisons between age‐associated proteins in the literature and Chronological Age‐ or Disease‐Associated Proteins, and FAM‐CPH APs.
**Table S4a:**. Combined list of disease‐associated proteins from the literature.
**Table S4b:**. Selected panels of disease‐associated proteins from the literature.
**Table S5:**. SASP‐associated proteins from the literature.
**Table S6:**. Annotated list of 13 identified novel biological aging‐associated proteins.
**Table S7:**. Cluster assignment of all 5416 measured proteins.
**Table S8:** Correlations between the two proteins robustly associated with mortality and aging‐related measures.

**Appendix S1:** acel70469‐sup‐0003‐AppendixS1.pdf.

## Data Availability

The data that support the findings of this study are available on reasonable request from the corresponding author. The data are not publicly available due to regulations set out by the Danish Data Protection Agency.
