# Supplementary material for: Plasma Proteomic Profiling of Young and Older Adults Identifies Candidate Biomarkers of Biological Aging at the Intersection of Age and Disease
Source: Aging Cell. 2026 Apr 3;25(4):e70469. doi: 10.1111/acel.70469 (PMC13052327; doi:10.1111/acel.70469)
Supplement: Supplementary file 1 — Figure S1: Overview of analyses performed in the study. Figure S2: Boxplots, dot plots, and violin plots for proteins with the highest differential expression between Older Controls and Young Controls representing “Chronological Age‐Associated Proteins”. Plots show median and interquartile range for each group: Young Controls (YC, green), Older Controls (OC, yellow), and Older Patients (OP, red). Boxplots display median and interquartile range, and whiskers extending to 1.5 times the IQR. Figure S3: Boxplots, dot plots, and violin plots for the 20 proteins with the highest differential expression between Older Patients and age‐matched Older Controls representing Disease‐Associated Proteins. Plots show median and interquartile range for each group: Young Controls (YC, green), Older Controls (OC, yellow), and Older Patients (OP, red). Boxplots display median and interquartile range, and whiskers extending to 1.5 times the IQR. Figure S4: Scree plot of dendrogram agglomerations for the main cluster analysis presented in the paper using Euclidean distance as dissimilarity measure. Figure S5: Normalized stability score of the Euclidean distance‐based hierarchical clustering with complete linkage by number of clusters based on the Rand index for sampling proportion 𝜈 = 0.95, 0.90, and 0.85. The normalized stability score was estimated using normalization by random labels. Figure S6: Scree plot of dendrogram agglomerations for the cluster analysis using Manhattan distance as dissimilarity measure. Figure S7: Stability assessment of protein selection procedure based on regularized Cox regression with LASSO penalty. The entire selection procedure was repeated across 1000 stratified bootstrap samples. The proteins presented were nonzero within a 1‐standard‐error range of the optimal model with the lowest partial likelihood deviance based on the full dataset. The blue color indicates nonzero proteins selected in the optimal model. Left: Boxplots of the 1000 bootstrap realizati [file ACEL-25-e70469-s003.docx]

**Supplementary Figures**


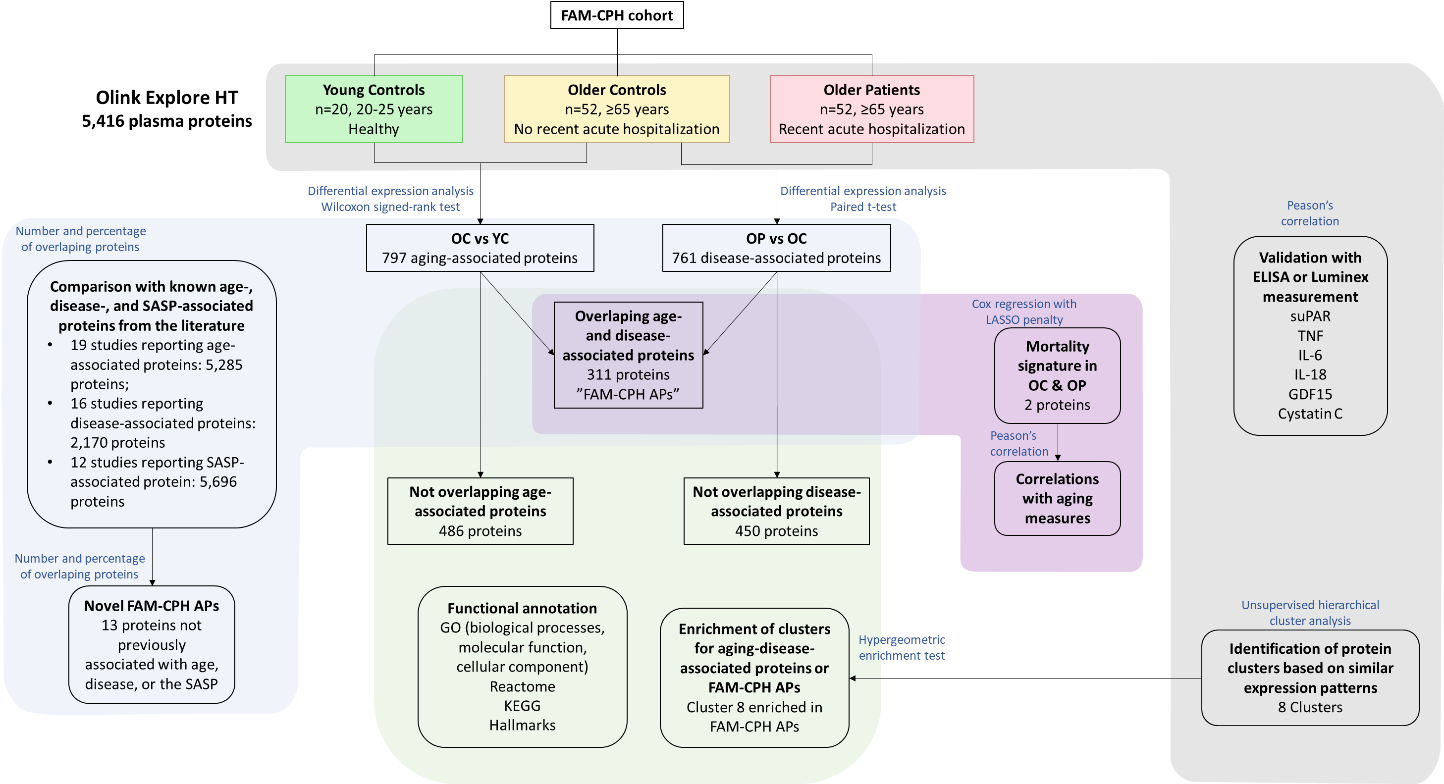


**Supplementary Figure 1.** Overview of analyses performed in the study.

Abbreviations: GDF15: growth differentiation factor 15; IL: interleukin; OC: Older Controls; OP: Older Patients; SASP: senescence-associated secretory phenotype; suPAR: soluble urokinase plasminogen activator receptor; TNF: tumor necrosis factor; YC: Young Controls.

**Supplementary Figure 2. Boxplots, dot plots, and violin plots for proteins with the highest differential expression between Older Controls and Young Controls representing “Chronological Age-Associated Proteins”.** Plots show median and interquartile range for each group: Young Controls (YC, green), Older Controls (OC, yellow), and Older Patients (OP, red). Boxplots display median and interquartile range, and whiskers extending to 1.5 times the IQR.

**Supplementary Figure 3. Boxplots, dot plots, and violin plots for the 20 proteins with the highest differential expression between Older Patients and age-matched Older Controls representing Disease-Associated Proteins.** Plots show median and interquartile range for each group: Young Controls (YC, green), Older Controls (OC, yellow), and Older Patients (OP, red). Boxplots display median and interquartile range, and whiskers extending to 1.5 times the IQR.


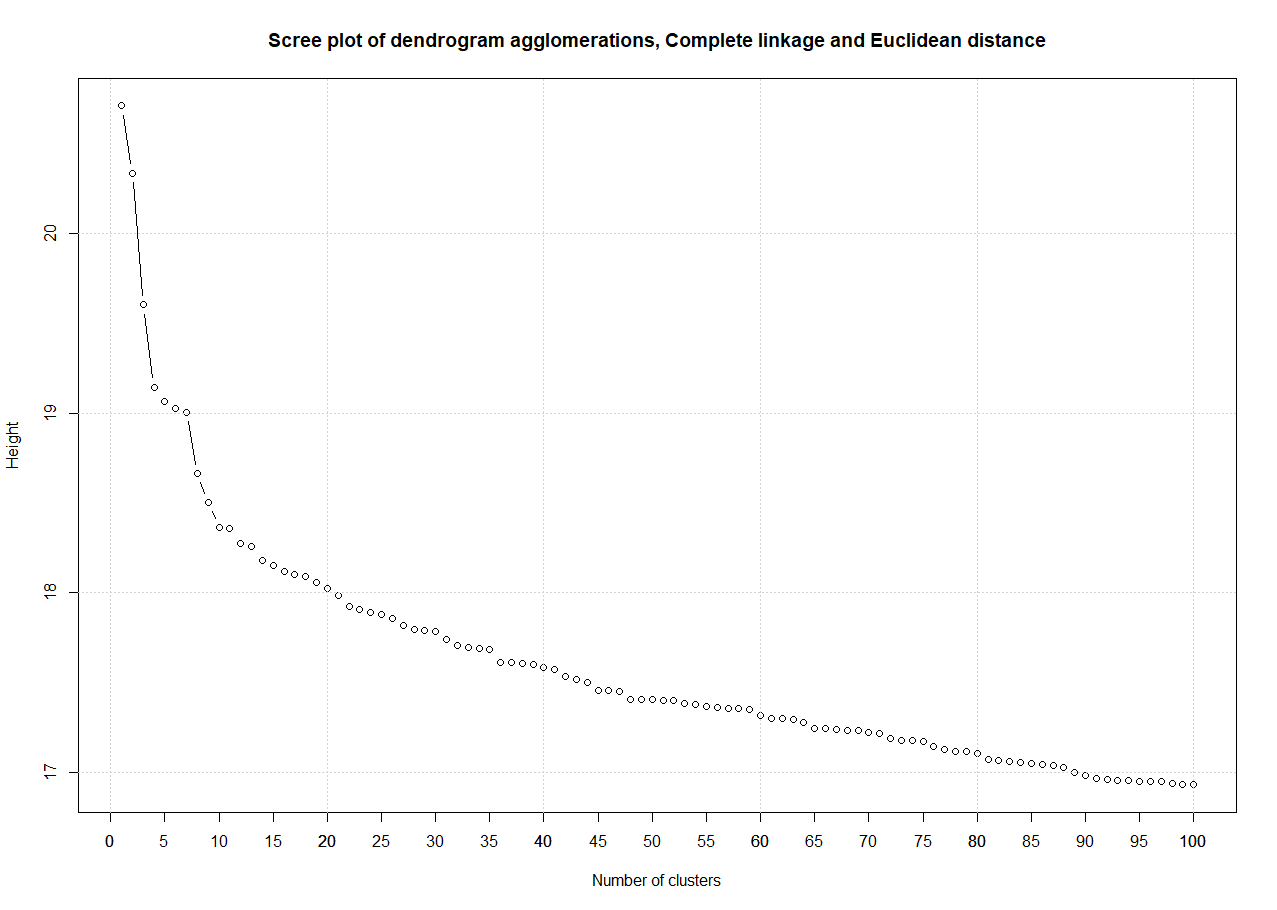


**Supplementary Figure 4.** Scree plot of dendrogram agglomerations for the main cluster analysis presented in the paper using Euclidean distance as dissimilarity measure.

**
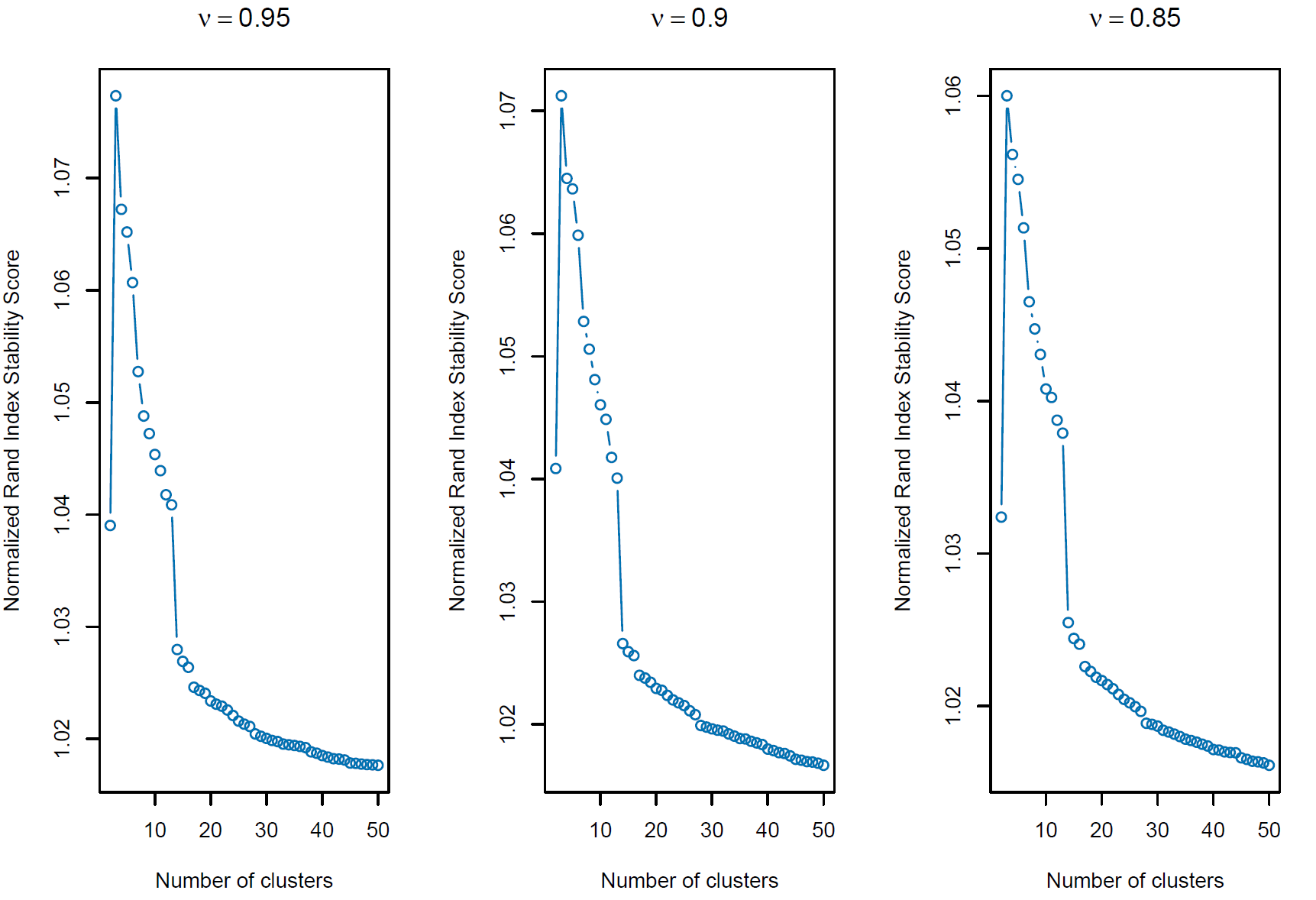
**

**Supplementary Figure 5.** Normalized stability score of the Euclidean distance-based hierarchical clustering with complete linkage by number of clusters based on the Rand index for sampling proportions $\nu=$ 0.95, 0.90 and 0.85. The normalized stability score was estimated using normalization by random labels.

**
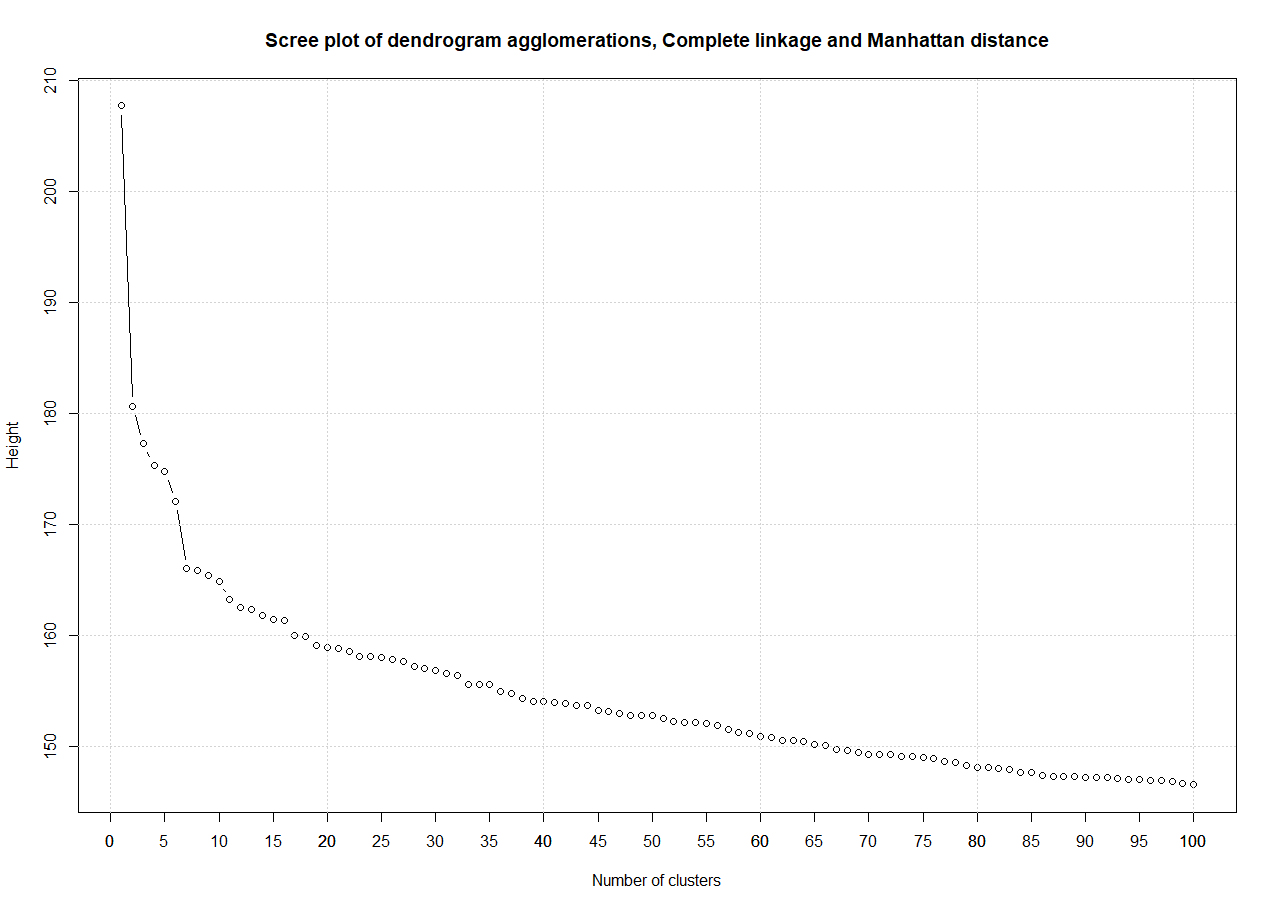
**

**Supplementary Figure 6.** Scree plot of dendrogram agglomerations for the cluster analysis using Manhattan distance as dissimilarity measure.

**Supplementary Figure 7. Stability assessment of protein selection procedure based on regularized Cox regression with LASSO penalty.** The entire selection procedure was repeated across 1000 stratified bootstrap samples. The proteins presented were nonzero within a 1-standard-error range of the optimal model with the lowest partial likelihood deviance based on the full dataset. The blue color indicates nonzero proteins selected in the optimal model. Left: Boxplots of the 1000 bootstrap realizations of the coefficients of the presented proteins. Right: Proportion of times the coefficient is zero in the bootstrap distribution.
