# Supplementary material for: Plasma Proteomic Profiling of Young and Older Adults Identifies Candidate Biomarkers of Biological Aging at the Intersection of Age and Disease
Source: Aging Cell. 2026 Apr 3;25(4):e70469. doi: 10.1111/acel.70469 (PMC13052327; doi:10.1111/acel.70469)
Supplement: Supplementary file 3 — Appendix S1: acel70469‐sup‐0003‐AppendixS1.pdf. [file ACEL-25-e70469-s002.pdf]

AGING CELL AUTHOR CHECKLIST. ***Authors should submit this checklist together with their manuscript. Please ensure that you have read the Author Guidelines in detail before submission.***

**Title**	Plasma proteomic profiling of young and older adults identifies candidate biomarkers of biological aging at the intersection of age and disease
**Authors**	Juliette Tavenier, Nikolaj Normann Holm, Thomas Kallemose, Morten Baltzer Houlind, Aino Leegaard Andersen Line Fleischer Hach, Magnus Berglind, Ove Andersen, Jan O. Nehlin, Line Jee Hartmann Rasmussen
**Manuscript Type**	Research article
**Total Character Count (including spaces)[1]**	55,717
**Word count of Summary[2]**	232
**Number of papers cited in the References[3]**	97
**Listing of all Tables (Table1, Table 2 etc)[4]**	Table 1

**Figure specifications (please complete one row per figure)[5]** ***Figure no.***	Colour ***(yes/no)***	Greyscale ***(yes/no)***	Black and white ***(yes/no)***	Single column (80mm) ***(yes/no)***	Double column (180mm) ***(yes/no)***	Size of figure at full scale (mm x mm) ***(insert details)***	Smallest font size used in the figure at full scale (minimum 6pt) ***(insert details)***
Figure 1	Yes	No	No	Yes	No	80 x 50	
Figure 2	Yes	No	No	No	Yes	180 x 140	
Figure 3	Yes	No	No	Yes	No	80 x 122	
Figure 4	Yes	No	No	No	Yes	180 x 120	
Figure 5	Yes	No	No	No	Yes	180 x 60	

[1] The maximum character count allowed is 50,000 (incl. spaces) for Primary Research Papers and Reviews, 10,000 for Short Takes.

[2] Summary should not exceed 250 words.

[3] Primary Research Papers can contain a maximum of two tables. If more are needed they should replace some of the Figures or can be placed in the Supporting Information.

[4] A maximum of 45 references is allowed for Primary Research Papers and 20 references for Short Takes.

[5] A Primary Research Paper may contain up to 6 figures and a Short Take up to 2 figures. Authors are encouraged to provide figures in the size they are to appear in the journal and at the specifications given.
